# Dual-Color Fluorescence *In Situ* Hybridization Reveals an Association of Chromosome 8q22 but Not 8p21 Imbalance with High Grade Invasive Breast Carcinoma

**DOI:** 10.1371/journal.pone.0070790

**Published:** 2013-07-25

**Authors:** Logan C. Walker, Margaret McDonald, J. Elisabeth Wells, Gavin C. Harris, Bridget A. Robinson, Christine M. Morris

**Affiliations:** 1 Cancer Genetics Research Group, Department of Pathology, University of Otago, Christchurch, New Zealand; 2 Mackenzie Cancer Research Group, Department of Pathology, University of Otago, Christchurch, New Zealand; 3 Department of Public Health and General Practice; University of Otago, Christchurch, New Zealand; 4 Department of Anatomical Pathology, Canterbury Health Laboratories, Christchurch, New Zealand; Health Canada and University of Ottawa, Canada

## Abstract

We previously reported molecular karyotype analysis of invasive breast tumour core needle biopsies by comparative genomic hybridization (CGH) and fluorescence in situ hybridization (FISH) (Walker et al, Genes Chromosomes Cancer, 2008 May;47(5):405-17). That study identified frequently recurring gains and losses involving chromosome bands 8q22 and 8p21, respectively. Moreover, these data highlighted an association between 8q22 gain and typically aggressive grade 3 tumors. Here we validate and extend our previous investigations through FISH analysis of tumor touch imprints prepared from excised breast tumor specimens. Compared to post-surgical tumor excisions, core needle biopsies are known to be histologically less precise when predicting tumor grade. Therefore investigating these chromosomal aberrations in tumor samples that offer more reliable pathological assessment is likely to give a better overall indication of association. A series of 60 breast tumors were screened for genomic copy number changes at 8q22 and 8p21 by dual-color FISH. Results confirm previous findings that 8p loss (39%) and 8q gain (74%) occur frequently in invasive breast cancer. Both absolute quantification of 8q22 gain across the sample cohort, and a separate relative assessment by 8q22:8p21 copy number ratio, showed that the incidence of 8q22 gain significantly increased with grade (p = 0.004, absolute and p = 0.02, relative). In contrast, no association was found between 8p21 loss and tumor grade. These findings support the notion that 8q22 is a region of interest for invasive breast cancer pathogenesis, potentially harboring one or more genes that, when amplified, precipitate the molecular events that define high tumor grade.

## Introduction

Despite recent advances in our understanding of the molecular basis of breast cancer, classical histological grading of breast cancer remains prominent in routine histopathological practice [1], [2], [3]. This is because pathological assessment of tumour grade offers a rapid and relatively inexpensive measure of tumor cell proliferation, differentiation and overall disease aggressiveness, assisting the clinical ascertainment of risk of recurrence and choice of adjuvant therapies through such algorithms as the Nottingham Prognostic Index [4]. Histological grade is established after microscopic evaluation of paraffin-embedded haematoxylin and eosin stained sections, and is typically represented by nuclear morphology, the number of mitoses and the degree of tubule formation. Patients with well differentiated (grade 1) tumors have significantly better survival than patients with poorly differentiated (grade 3) tumors [5]. Although routinely applied, issues of interobserver variability in the assessment of histological grade are a well recognised and ongoing challenge [6], [7], [8], [9], [10]. There is a need for advancement in the accuracy and reproducibility of routinely applied histopathological tools for better refined breast cancer diagnosis and prognosis.

Genome-wide profiling technologies have contributed much to current understanding of the association between breast cancer genotype and phenotype. Information learned from these technologies is progressively challenging the way in which conventional pathology protocols are applied, with an overall objective to stratify breast cancer patients at the time of diagnosis into more effective clinical risk groups for better targeted treatment interventions [1], [2], [3], [11], [12], [13]. However, the clinical translation of newly identified biologically relevant gene markers, including screening methods to allow their detection, in many cases requires further validative research.

Since development in 1992 by Kallioniemi and colleagues [14], metaphase comparative genomic hybridization (mCGH) and subsequent high-resolution array CGH (aCGH) adaptations [15], [16] have been widely applied to the interrogation of genomic copy number imbalances as they occur in breast cancer. Recurrent patterns of chromosomal loss and gain have been shown to associate with different histopathological subtypes, and their functional relevance assessed in turn by correlation with global gene expression signatures [12], [17], [18], [19], [20], [21], [22], [23], [24]. Amplification of discrete genomic regions in human breast carcinomas highlighted by these methodologies has resulted in the refined characterization of specific genes associated with breast tumourigenesis, including *MYC* at 8q24, *CCND1* at 11q13 and *ERBB2* at 17q12 [24], [25], [26], [27].

Our previously reported mCGH analysis of 42 diagnostic core needle biopsies from primary invasive carcinoma of the breast showed that copy number gain or amplification involving chromosome 8q occurred in 64% of all tumors, and selectively in 84% of grade 3 tumors [28]. These findings are in agreement with previous reports using mCGH that identified 8q gain in 41–65% of post-surgical breast tumor specimens [29], [30], [31], [32], [33], [34], [35], [36], [37], [38], [39], [40], [41], [42], and in 68%–90% of grade 3 tumors [33], [34], [37], [40]. The patterns of 8q gain are not identical between different tumors, with some regions affected more frequently than others. Our study identified a recurring region of gain on chromosome 8 at band q22 (8q22), a finding of interest because it overlaps with common regions of gain reported independently by others, including 8q21–q23 [35], 8q21-qter [36], [37], [38], [39], [40], [41], [42], 8q22-qter [32] and 8q22–q23 [29], [31], [34], [41]. Higher resolution microarray studies using BAC and oligonucleotide platforms have in some cases allowed better definition of the precise genomic co-ordinates of regions of 8q gain, although the widely differing platforms, sampling characteristics and statistical algorithms for data analysis that have been applied have made consensus difficult [19], [20], [21], [22], [23], [43], [44], [45], [46], [47], [48], [49], [50]. Copy number gain at 8q22 has also been shown to correlate with increased expression levels of several genes across this region, including *MTDH, LAPTM4B* and *YWHAZ*, and associated with poor clinical outcome for breast cancer patients [51], [52].

In contrast to 8q, the short arm of chromosome 8 (8p) is reported to show copy number loss in breast tumors of high grade or in association with worse prognosis. However, the frequency of 8p loss is lower than for 8q gain overall (range 18–44%) [28], [30], [31], [32], [33], [34], [35], [36], [37], [38], [39], [40], [41], [42], and in the high-grade tumor subsets (range 42–74%) [28], [33], [34], [37]. mCGH studies have identified various minimal regions of deletion, including 8p12-pter [31], 8p21-pter [42], 8p22-pter [34], [36], 8p21–p22 [28], [38] and 8p22–p23 [35], [40], [41]. Subsequent high-resolution profiling studies have confirmed that loss of 8p, from 8p12 to the telomere, sometimes with proximal amplification of 8p11–12, constitute the most frequent copy number alterations [22], [50], [53].

Our earlier described mCGH study of diagnostic core biopsies suggested significant association between 8q22 gain, 8p21 loss and grade 3 breast cancer. Subsequent FISH analysis of tumor touch imprints prepared from a subgroup of the same biopsies supported these findings [28]. For the present study, tumor touch imprints were prepared from a different larger cohort of 60 post-operative invasive breast tumor specimens, which offer the advantage of more precise scoring of histological grade from corresponding paraffin sections. Results confirm significant association of 8q22 gain with high-grade breast cancer, reinforcing need for future focussed interrogation of genomic features in this region.

## Materials and Methods

### Ethics Statement

All patients provided written consent to gift their tumor tissue to the Cancer Society Tissue Bank for future research. The use of banked samples for this research was approved by the Canterbury Ethics Committee.

### Breast Tumor Samples

Tumor touch imprints were prepared from resected tumor samples of 60 breast cancer patients presenting for surgery at Christchurch Hospital. Pathological review was carried out as described previously [28]. This assessment includes macroscopic and microscopic measurement of tumor size, and grading according to a modified Bloom and Richardson protocol [5]. Immunohistochemistry was applied to determine hormone receptor status (ESR1, PGR) [H-score] [54], [55] and ERBB2 (HER-2) status (HercepTest, Dakocytomation, Glostrup). Any lymph nodes removed during surgery were examined macroscopically and microscopically for metastatic tumor infiltration.

### Tumor Touch Slide Imprints

Touch slide imprints were prepared by briefly blotting surgically excised tissue on lint-free paper to remove excess moisture and then firmly pressing tissue samples against four separate pre-cleaned microscope slides. Cells were air dried and then fixed by soaking the slides in methanol:acetic acid (3∶1) for 20 minutes, air dried and stored at −20°C with desiccant.

### Fluorescence in situ Hybridization

Bacterial artificial chromosome (BAC) DNA probes RP11-177H13 (8p21) and RP11-10G10 (8q22) contain sequences that map to human chr8:23,051,329-23,230,640 and chr8:101,209,852-101,362,596 (GRCh37/hg19 build), respectively, and were selected from a replicated 1 Mb Wellcome Trust Sanger Institute clone set [56] based on their central location within the 8p21 and 8q22 cytobands of interest [28]. RP11-177H13 spans coding domains of several genes, including *TNFRSF10A*, *CHMP7*, *R3HCC1* and *LOXL2*. BAC RP11-10G10 contains *SPAG1* and *RNF19A*. These two BAC clones were selected because of their central location within frequently lost or gained regions of interest at cytobands 8p21 and 8q22, respectively [28]. FISH methods, including probe preparation, slide preparation, hybridization and post-hybridization washes were performed as described [28]. Briefly, cells from tumor touch imprints were treated with pepsin, paraformaldehyde fixed, and genomic DNA was denatured by immersion for 4 min at 72–74°C in a solution of 70% formamide/2×SSC (pH 7.0) immediately prior to hybridization. BAC probes RP11-177H13 and RP11-10G10 were labeled by nick-translation using Spectrum Red and Spectrum Green (Vysis, Downers Grove, IL, USA), respectively. Labelled probes were hybridized at a concentration of 40–50 ng/µl in a 10 µl mix containing 50% formamide, 2×SSC, 10% dextran sulphate, 2 µg/µl herring sperm DNA and 1.5 µg/µl Cot-1 DNA. The probe mix was denatured at 75°C for 5 min, and allowed to preanneal at 37°C for 30 min, then immediately applied, under coverslip, to the denatured touch preparations and hybridized overnight at 37°C in a humidified chamber. Post-hybridization washes were carried out by washing two times, 5 min each, in 2×SSC (pH 7) at 45°C, followed by two 5 min washes in 50% formamide/2×SSC (pH 7) at 45°C, then two further washes, 5 min each, in 2×SSC at 45°C, and a final 15 min wash in 0.1×SSC at 60°C.

### FISH Data Acquisition and Analysis

Following post-hybridization washes, slides were mounted in glycerol containing the antifade agent *p*-phenylene-diamine dihydrochloride (Sigma, 1 mg/ml) and 0.25 mg/ml DAPI (4′,6-diamidino-2-phenylindole) as counterstain for G-band visualization. Fluorescence images from interphase cells were digitally captured at selective bandwidths using a Leitz Aristoplan microscope equipped with a Photometrics KAF1400 CCD camera and QUIPS Smartcapture software (version 1.3; Vysis Inc, Downers Grove, IL). Areas with well-separated nuclei and overall good hybridization signal were selected for analysis by fluorescent microscopy. For each case, red (RP11-177H3, 8p21) and green (RP11-10G10, 8q22) fluorescent signals were counted at 100× magnification in at least 50 non-overlapping interphase nuclei. Two different approaches were applied to define chromosomal copy number changes. In the first analysis, absolute copy number gains and losses of 8p21 and 8q22 were scored as positive if more than 10% of analyzable nuclei harbored a copy number change. A signal score of 12 was assigned to nuclei displaying amplification of 8q22 when FISH signals were unable to be precisely enumerated. In the second approach, relative copy number for 8q22 was determined by calculating the mean copy number for RP11-10G10 specific signals and dividing by the respective value for RP11-177H13 at 8p21. To avoid the possibility that a greater green to red signal ratio was due solely to a deletion of chromosome 8p21, chromosome 8q22 gain was defined as relative 8q22/8p21 signal ratio greater than 2.0.

### Statistical Analysis

For cross-tabulation of categorical variables two-sided mid-p Fisher's exact test was used or, where appropriate, the Extended Mantel-Haenzel chi square test for linear trend[57]. Survival analyses were carried out in SAS 9.2 (www.SAS.com) using the log-rank test, with and without stratification, or proportional hazards models.

## Results

### Patient Sample Characteristics

Tumor histopathology of the 60 breast tumor samples imprinted for FISH analysis in this study is summarized in Supporting Information Table S1 along with patient lymph node status. Fifty six of the 60 tumors were histopathologically classified as IDC and four were classified as invasive lobular carcinoma (ILC) (Supporting Information Table S1). Fifteen samples were classified as grade 1, 16 as grade 2, and 29 as grade 3. Tumor immunophenotype was determined for ESR1 (44 positive, 16 negative), PGR (35 positive, 25 negative), and ERBB2 (19 scored as negative or 1+, 11 scored as 2+, 11 scored as 3+ and 19 were not assayed). Lymph node status was confirmed in 56 samples, and 28 of these patients were found to have lymphatic spread of the disease (Supporting Information Table S1). Of the 15 grade 1 tumors, all were ESR1 positive and one of 10 tumors examined for ERBB2 expression showed 3+ staining. In comparison, 15 of the 29 grade 3 tumors (52%) were ESR1 negative and eight of the 24 tumors analysed for ERBB2 expression (33%) were characterized by 3+ staining.

### Assessment of 8p21 and 8q22 Copy Number Status

BAC clones RP11-177H13 and RP11-10G10 hybridized cleanly to tumor nuclei in 54 of 60 breast tumor touch preparations. Data derived from the FISH analysis are detailed in Supporting Information Table S2 and summarized in Table 1. Of the 54 samples, the total cells analyzed ranged from 50 to 232 (Table 1). For the remaining six preparations (BT70, BT80, BT93, BT144, BT150 and BT172), analyzable cells were <50 (range 15–44) (Supporting Information Table S2), and low signal intensity was a complicating factor in two of these samples (BT93 and BT172). Data corresponding to the latter six tumors were therefore excluded from further analysis.

**Table 1:** Summary of signal patterns observed after FISH of the 8p21- and 8q22-band specific BAC probes, RP11-177H13 and RP11-10G10, respectively, to 54 breast tumor touch preparations.

Case	Histological Type	Grade	Total cells analysed	Nuclei counts				8q22/8p21 ratio
				>2 copies of 8p21	<2 copies of 8p21	>2 copies of 8q22	<2 copies of 8q22	
BT9	IDC	1	103	0	81	5	2	1.68
BT38	IDC	1	202	25	0	25	0	1.00
BT39	IDC	1	206	11	1	5	8	0.97
BT43	IDC	1	104	3	1	2	25	0.88
BT45	IDC	1	100	0	82	0	15	1.57
BT63	IDC	1	123	0	14	83	0	2.40
BT66	IDC	1	95	53	0	53	0	1.00
BT67	IDC	1	216	0	0	5	1	1.01
BT69	IDC	1	203	3	0	3	0	1.00
BT83	IDC	1	111	103	0	103	0	1.00
BT207	IDC	1	63	4	5	62	0	3.88
BT7*	IDC	2	115	3	46	107	0	3.36
BT19	IDC	2	102	98	0	99	0	1.03
BT44	IDC	2	211	37	0	34	0	0.99
BT52	IDC	2	161	31	0	36	0	1.01
BT56*	IDC	2	122	1	94	110	0	2.66
BT68	IDC	2	154	0	137	8	1	1.85
BT71	IDC	2	123	48	1	122	0	3.31
BT109	IDC	2	116	94	0	95	0	1.06
BT146	IDC	2	102	0	92	2	0	1.84
BT173	IDC	2	152	2	0	122	0	3.20
BT193	IDC	2	207	2	35	1	5	1.08
BT208*	IDC	2	111	0	91	107	0	2.89
BT215	IDC	2	109	66	0	66	0	1.00
BT2	IDC	3	108	59	3	74	1	3.35
BT5	IDC	3	102	1	7	65	1	1.37
BT30	IDC	3	142	50	48	118	0	3.08
BT37*	IDC	3	232	0	215	218	4	3.16
BT40	IDC	3	111	3	0	109	0	3.05
BT42*	IDC	3	114	1	98	98	0	2.59
BT46	IDC	3	50	2	1	21	3	1.24
BT47*	IDC	3	104	0	77	89	0	2.59
BT57	IDC	3	111	129	2	87	0	1.59
BT59	IDC	3	122	1	106	114	0	3.52
BT61*	IDC	3	128	1	107	115	0	2.55
BT72	IDC	3	109	12	4	89	0	3.77
BT76	IDC	3	104	4	0	84	0	4.76
BT78	IDC	3	206	1	1	5	0	1.02
BT91	IDC	3	113	3	0	86	0	1.62
BT107	IDC	3	77	19	0	20	2	1.07
BT108*	IDC	3	102	2	66	50	0	2.04
BT117	IDC	3	128	7	36	91	0	2.33
BT119*	IDC	3	161	0	64	75	0	1.62
BT120	IDC	3	109	0	70	82	0	5.93
BT139	IDC	3	89	2	0	2	0	1.00
BT158	IDC	3	105	5	1	101	0	4.64
BT182*	IDC	3	130	1	58	109	1	2.33
BT184	IDC	3	101	1	0	76	0	1.73
BT188	IDC	3	112	0	93	97	0	3.95
BT190	IDC	3	102	3	2	4	0	1.02
BT191	IDC	3	101	0	0	98	0	1.98
BT55	ILC	1	78	0	5	2	76	0.57
BT124	ILC	2	151	1	0	1	0	1.00
BT31	ILC	3	119	116	0	119	0	1.16

Abbreviations: IDC, invasive ductal carcinoma; ILC, invasive lobular carcinoma. Cases with likely i(8q) are marked with an asterisk.

**Figure 1:**
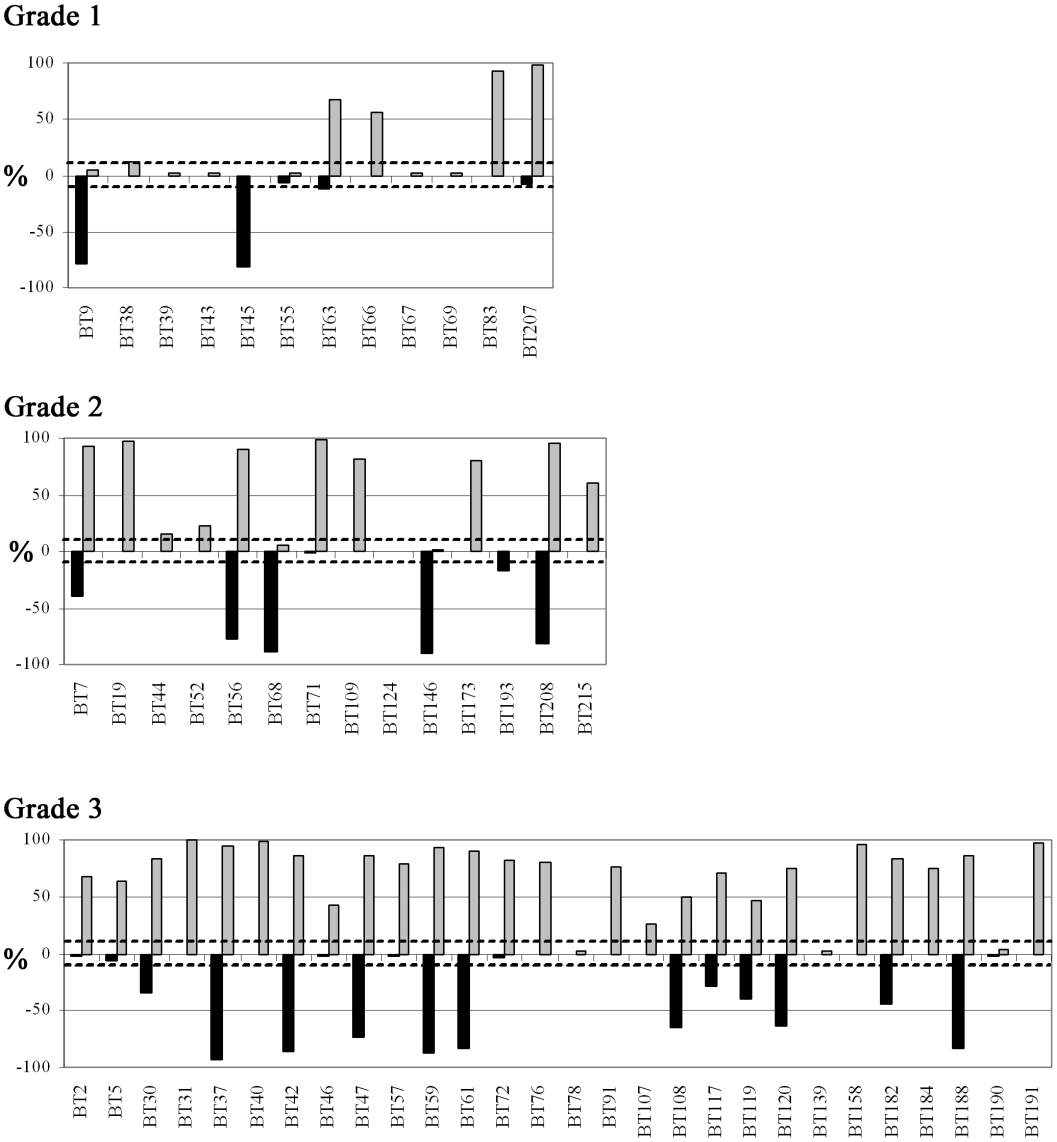
Proportion of nuclei from 54 breast tumor touch preparations after FISH showing decreased copy number of BAC probe RP11-177H13 (8p21; black bars), and/or increased copy number of RP11-10G10 (8q22; gray bars). Copy number changes are marked as loss or gain if at least 10% (marked by dashed lines) of nuclei show less than 2 signals from RP11-177H13 or more than 2 signals from RP11-10G10. Tumors are sorted into three graphs according to histological grade.

**Figure 2:**
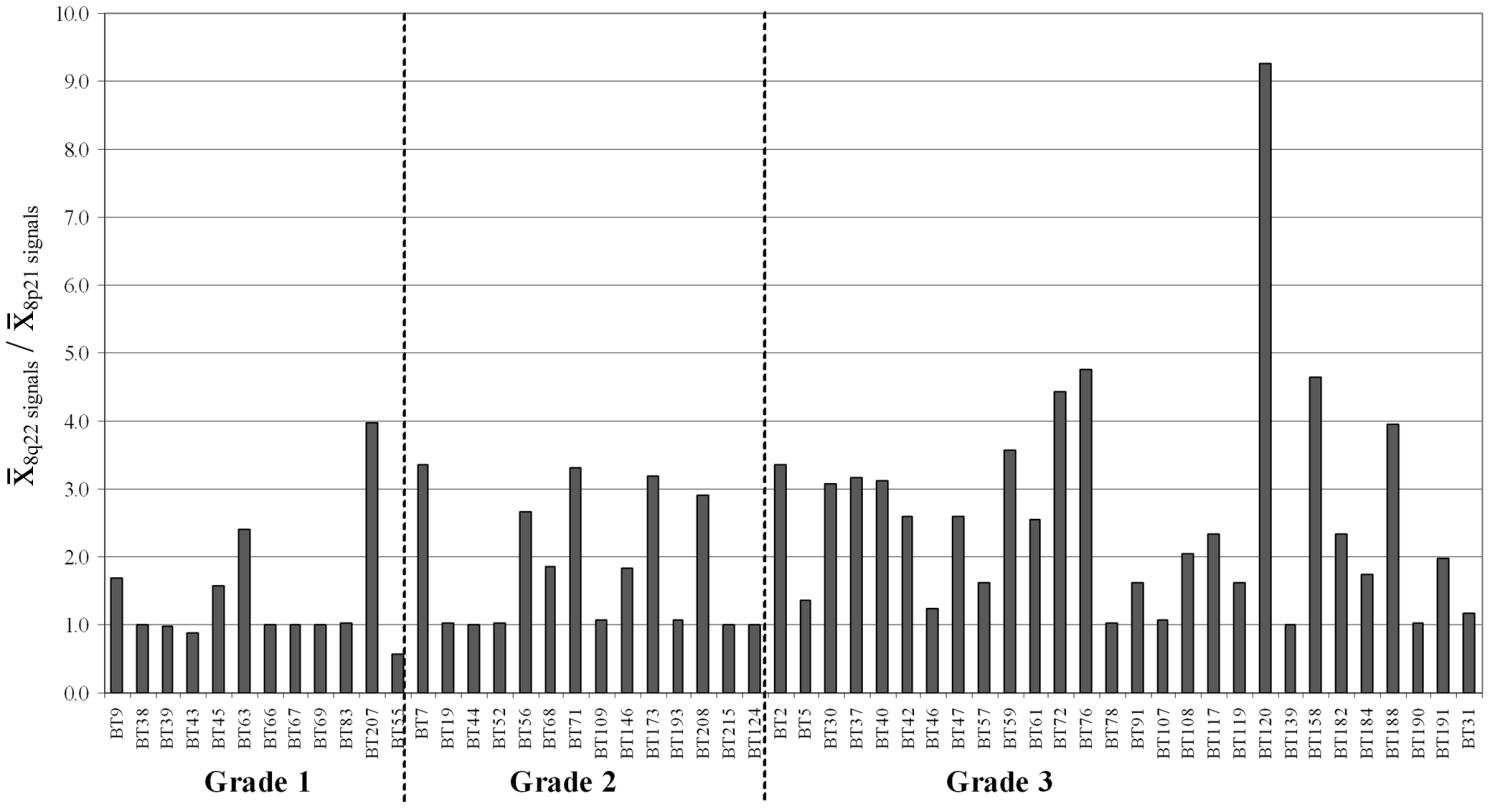
Relative copy number of 8q22 in 54 breast tumor touch preparations categorized by histological grade. Relative copy number for 8q22 was determined by calculating the mean copy number for RP11-10G10 (8q22) specific signals and dividing by the respective value for RP11-177H13 at 8p21. Copy number gain is scored positive by a ratio greater than 2.0.

A representative example of signal patterns observed in interphase nuclei of the remaining 54 samples is shown in Supporting Information Figure S1. Absolute gain or loss of 8q22 (RP11-10G10) was detected in 74% (40/54) or 6% (3/54) of these tumors, respectively (Fig. 1). Thirty one percent (17/54) of tumors analyzed also harbored a high level gain of 8q22 (more than 5 copies in greater than 10% of nuclei scored) (Supporting Information Table S2). By comparison, assessment based on 8q22:8p21 copy number ratio revealed 8q22 gains in 43% (23/54) of tumors (Table 1 and Fig. 2).

Absolute chromosomal gain or loss at 8p21 (RP11-177H13) was detected in 28% (15/54) or 39% (21/54) of the tumors, respectively (Fig. 1). All 15 tumors showing a gain at 8p21 also harbored a gain at 8q22, although we note variability in the number of nuclei scored for each of these chromosomal imbalances (Table 1). For 11 of these 15 tumor samples, 8q22:8p21 signal ratios <2.0 (range 1.0–1.6; mean 1.1) were consistent with whole chromosome 8 gain, and in nine of these cases (BT19, BT31, BT38, BT44, BT52, BT66, BT83, BT109, BT215), absolute copy number status including number and distribution of 8p21 and 8q22 signals across total cells analyzed supported this interpretation (Supporting Information Table S2, Table 1).

None of the tumors analyzed showed mixed populations of cells having both loss and gain of either 8p21 or 8q22. The formation of an isochromosome involving 8q with one remaining copy of chromosome 8, or alternatively unbalanced translocation(s) or isoderivatives resulting in 8p loss, was suggested in 10 tumor samples (BT7, BT37, BT42, BT47, BT56, BT61, BT108, BT119, BT182 and BT208) for which 31% to 79% of cell nuclei exhibited three copies of 8q22 but only one copy of 8p21 (Supporting Information Table S2). All specimens showing unbalanced 8p/8q signal patterns were scored as higher grade breast tumors (n = 3 grade 2; n = 7 grade 3). A tetraploid (4n) equivalent of the i(8q) signal pattern consistent with two copies of 8p21 and six copies of 8q22 was present in 10–50% of nuclei analyzed in four of these 10 samples (BT7, BT47, BT56 and BT182), and the latter pattern was also observed in samples BT40 and BT63 (Supporting Information Table S2).

For 52 of the 54 cases analyzed in detail, varying numbers of cells showed two signals only for each of the RP11-177H13 and RP11-10G10 probes applied, consistent with normal 8p21 and 8q22 copy number status. In 36 of these 52 cases, the proportion of nuclei that displayed this normal signal pattern ranged from 10%–99% (Supporting Information Table S2). For six cases, normal signal patterns were found in greater than 90% of cells analyzed, whereas in 12 cases these patterns were observed in less than 10% of cells analyzed (Supporting Information Table S2).

### 8p21 and 8q22 Copy Number Aberrations and Tumor Pathology

Copy number changes involving 8p21 and 8q22 were correlated with tumor pathology and the results are presented in Table 2. When tumors were grouped according to histological grade, the incidence of absolute and relative 8q22 gain significantly increased with grade (p = 0.004 and p = 0.02, respectively) (Table 2). In contrast, there was no association between histological grade and absolute 8p21 copy number change (Table 2). The proportion of tumors that harbored more than five copies of 8q22 was also higher in grade 3 tumors (11/28, 40%) when compared to grade 2 (4/14, 29%) and grade 1 (2/12, 17%) tumors (Supporting Information Table S2). Of the 10 cases with suggested i(8q)/ider(8q)/t(8q;?), 3/10 were classified as grade 2, and 7/10 were classified grade 3 (Supporting Information Table S2). Of six cases showing signal patterns consistent with duplication of one or other of these structural 8q imbalances, one was grade 1, two were grade 2 and three were grade 3 (Supporting Information Table S2, Table 1). In contrast, a higher proportion of grade 1 (3/12; 25%) or grade 2 tumors (5/14, 36%) than grade 3 tumors (1/28; 4%) showed 8q22:8p21 ratio and absolute copy number status consistent with gain of one or more copies of chromosome 8 (Supporting Information Table S2, Table 1). No significant association was seen between copy number changes at 8p21 or 8q22 and tumor type (IDC or ILC), ESR1 status, PGR status, ERBB2 status or nodal involvement (Table 2).

**Table 2:** Association of 8p21 loss and 8q22 gain with clinicopathologic and histologic features of 54 primary breast carcinomas.

Pathology feature			8p21 loss (number)		*P*	8q22 gain (number)		*P*	Relative 8q22 gain (number)		*P*
			−	+		−	+		−	+	
IDC		*n = 51*	30	21		12	39		28	23	
ILC		*n = 3*	3	0	0.22^a^	2	1	0.18^a^	3	0	0.18^a^
Grade	1	*n = 12*	9	3		7	5		10	2	
	2	*n = 14*	8	6		4	10		9	5	
	3	*n = 28*	16	12	0.64^b^	3	25	0.004^b^	12	16	0.02^b^
ESR1	Negative	*n = 16*	11	5		3	13		8	8	
	Positive	*n = 38*	22	16	0.48^a^	11	27	0.47^a^	23	15	0.49^a^
PGR	Negative	*n = 24*	16	8		5	19		12	12	
	Positive	*n = 30*	17	13	0.47^a^	9	21	0.47^a^	19	11	0.34^a^
ERBB2	0/1+	*n = 19*	13	6		4	15		11	8	
	2+	*n = 9*	8	1		2	7		8	1	
	3+	*n = 10*	3	7	0.13^b^	1	9	0.68^b^	3	7	0.38^b^
	unknown	*n = 16*									
LN	Negative	*n = 25*	16	9		8	17		16	9	
	Positive	*n = 25*	15	10	0.78^a^	5	20	0.36^a^	13	12	0.41^a^

a Two-sided mid-p Fisher's exact test;

b Extended Mantel-Haenszel chi square for linear trend.

### Survival Analysis

Analysis of survival time until death from breast cancer was assessed for the 54 cases from this study and an additional nine cases from our previous study [28] where follow-up data were available. Forty-seven of these 63 cases showed 8q22 gain. All patients were potentially observed for at least five years (Supporting Information Table S3). The hazard ratio for death from breast cancer for those with 8q22 gain was 0.67 (compared with loss/no change) but this was not significant (p = 0.29). Gain was more common at high grades (39%, 71%, 91% across grades 1–3, respectively (p = 0.0003), but stratification by grade had little effect on the comparison of gain or no-gain (p = 0.20). Patients without 8q22 gain tended to be older than those with gain: 72.1 years (SD = 11.7) compared with 60.2 years (SD = 11.7), p = 0.01 (Supporting Information Table S3). The p-values for gain versus no-gain were slightly higher after accounting for age whether by stratification into those under or over 65 (p = 0.36) or by inclusion of age in a proportional hazards model (p = 0.46). With only 32 deaths, further exploration of these two possible confounders (grade and age) was not possible.

## Discussion

Our previous interphase FISH analysis of core needle biopsy touch imprints had confirmed mCGH findings for the same sample set to show that 8q22 copy number changes occur frequently in breast cancer and are significantly associated with grade 3 tumors [28]. The present study extends this earlier work through application of FISH and an identical 8p21/8q22 probe set to tumor touch imprints prepared from a larger series of histologically more precise excised breast tumor biopsies. Results detailed here confirm that copy number changes affecting 8p and 8q occur frequently in invasive breast tumors. Moreover, this study adds new evidence to support an association between 8q22 copy number gain and grade 3 invasive carcinoma.

The 8q22 region was recently shown to harbor the metastasis gene, *MTDH*, which was overexpressed and amplified in poor-prognosis breast cancer [51]. Although characterization of MTDH revealed a function in both promoting metastasis and chemoresistance, Hu and colleagues did not explore a possible relationship between MTDH function and the development of grade 3 tumors. By comparison, Sotiriou *et al* explored the association of gene expression profiles with histological grade in a cohort of breast IDC tumors [58]. This study showed that 183 genes were expressed differently between 33 grade 1 and 31 grade 3 tumors. Interestingly, nine genes that mapped to 8q were significantly overexpressed in grade 3 tumors. Three of these nine genes (*RAD54B, CCNE2, POLR2K*) map as close as 113 kb (*POLR2K*) and up to almost 6 Mb (*RAD54B*) centromeric to the BAC probe RP11-10G10 used in this study. Previously, Pollack *et al* had shown that increased DNA copy number extending across an ∼11 Mb region within 8q21–q22, from *NBN* (*NBS1*) to *YWHAZ* (chr8:90,945,564-101,962,79), was associated with correspondingly altered mRNA levels for the genes found within the amplified region [19]. Although not represented on their array, both *CCNE2* and *RAD54B* were noted genes of interest mapping within this region [19], as does *MTDH*. Our findings reported here using BAC clone RP11-10G10 are therefore consistent with these earlier studies. Our findings also align well with studies using higher density array platforms for genome-wide copy number assessment that report increased incidence of 8q22 gain in association with different biological characteristics that are commensurate with higher-grade malignancy, including ESR1-negative status, and a luminal B rather than luminal A profile [21], [43], [47]. In contrast to the findings of Hu *et al* [51], we were unable to demonstrate an association between shorter survival time and 8q22 gain with our cohort of 63 breast cancer patients. However, it is important to note that the patient cohort used by Hu *et al* was significantly younger (<50 years) than that used in our study, suggesting that the prognostic implication of 8q22 gain may only be relevant for early onset breast cancer.

CGH studies, both conventional and array, of pre-cancerous ductal carcinoma in situ (DCIS) have shown that 8q gains, including the 8q22 region, occur during early stages of breast tumorigenesis, but that this aberration is particularly associated with higher-grade DCIS [46], [59], [60], [61], [62]. Based on different frequencies of 16q loss between grade 1 and grade 3 breast tumors, it has been suggested that low grade and high grade tumors may evolve through different mechanistic pathways [34], [37], [63]. Similarly for 8q, it is possible that early over-representation of 8q22 may be a predetermining factor of grade 3 invasive breast cancer early in tumor development. However, the observation in our present study that absolute or relative gains involving the 8q22 region were also observed in 42% of grade 1 tumors supports the notion that a large proportion of invasive tumors share this aberration independently of histological grade. This result is consistent with other reports that have also highlighted shared chromosomal aberrations between low and high grade tumors, suggesting a model that predicts a significant number of breast tumors showing progression through the grades [46], [50], [64], [65]. Further studies are therefore required to refine this model and to understand the relationship between 8q22 gain, other recurring chromosomal imbalances and different morphological breast tumor subtypes.

In contrast to 8q22 gain, loss of 8p21 did not correlate significantly with tumor grade in our present FISH study. These results differ from our earlier report using mCGH, which described significantly increased frequency of 8p loss in high-grade IDC, and also from the reports of others which showed a similar trend when correlations with histological grade were assessed [33], [34], [66]. The study of Armes et al compared CGH and high-resolution gene expression profiles of 53 breast tumor samples, and showed that 8p loss was associated with grade 3 and ESR1-negative tumors. Microarray profiling identified 22 genes that mapped to 8p and for which gene expression levels were significantly lower in the grade 3 tumors. Three of these genes were selected for FISH analysis, and of these, *PCM1* at 8p22 (∼5 Mb telomeric of BAC clone RP11-177H13) showed highest frequency of loss, affecting 45% of analyzable cases [66]. Using BAC arrays, Loo and coauthors have also reported increased incidence of 8p loss in ER-negative IDC compared with ER-positive IDC [43]. However, in a subsequent study, using Affymetrix GeneChip Mapping 10 K arrays but confined to ER-positive tumors, they reported that 8p loss occurs more frequently in IDC compared with ILC, and was prevalent in a higher grade ER-positive IDC subset [67]. Hwang and colleagues identified genomic regions within two BAC clones at 8p23.1 and 8p23.3 that separated recurrent from nonrecurrent IDC subgroups, but then found no difference in overall survival or systemic recurrence-free survival in the cohorts identified by these genomic segments [49]. In the same study, three of a total four regions showing loss in greater than 50% of the total 62 samples analyzed by aCGH mapped to 8p, including one region at 8p21.1–21.3 that overlaps BAC clone RP11-177H13 and spans *CHMP2* and *LOXL2* [49]. Thus, whereas 8p loss is clearly a significant and recurring feature of invasive breast cancer, there is much still to understand about its place in the hierarchy of genomic changes that accompany tumour initiation and progression.

In this study, 8q22 gain was determined using two methods: 1) scoring absolute signal frequency after FISH with RP11-10G10, and 2) calculating the mean FISH signal copy number ratio between RP11-10G10 and RP11-177H13 at 8p21. Although a correlation between 8q22 and grade 3 breast tumors was demonstrated using both methods, a higher frequency of absolute copy number gain was observed in grade 3 tumors (25/28) compared with relative 8q22 gain (16/28). One interpretation is that 8q22 copy number increase resulted from chromosome 8 polysomy in nine of the grade 3 tumors. However, gain of whole chromosome 8 was only apparent in one of these nine tumors as indicated by an 8q22:8p21 signal ratio less than 2.0 and a majority of abnormal cells showing identical signal copy numbers for the two FISH probes used. The discordancy shown in the remaining eight cases may be attributed to high numbers of contaminating normal cells and/or our conservative 8q22:8p21 ratio threshold for 8q22 gain.

FISH analysis of interphase nuclei from 10 tumors revealed sizeable cell populations showing simultaneous loss of chromosome 8p21 with duplication of 8q22. These signal patterns are consistent cytogenetically with presence of an isochromosome 8q, an isoderivative including 8q22, or unbalanced translocation(s) with breakpoints proximal to 8q22 incurring 8p loss. These and other structural aberrations of chromosome 8 have been reported at high frequency following detailed comparisons of mCGH profiles with multicolor spectral karyotyping (SKY) karyotypes in breast cancer cell lines [68], [69], [70]. Unbalanced 8p/8q signal patterns were more frequent in our grade 3 tumor subset, an observation consistent with the well recognized increasing genomic complexity of advanced malignancy. We note here that the various structural modalities by which 8q gain may arise would suggest that in some cases loss of 8p is mechanistically linked to these, and therefore a bystander to the biologically more impactful 8q gain. If the genes critical to tumor progression are located on 8q then the significance of genes deleted on 8p, at least in these cases, might be questioned.

In summary, using FISH analysis of tumor touch imprints prepared from excised breast tumor specimens, we have provided further evidence that a copy number gain at chromosome 8q22 is associated with typically aggressive grade 3 tumors. Because histological grade in breast cancer provides clinically important prognostic information, the association with copy number gain at 8q22 suggests the location of one or more genes or regulatory elements that may play a key role in determining grade and which may be candidate biological marker(s) for poor prognosis. Further research is necessary to identify such genes, which may lead to a better understanding of breast tumorigenesis as well as potential prognostic markers and novel targets for therapeutic intervention.

## Supporting Information

Figure S1 Supplementary Figure 1.
(DOC)

Table S1 Histopathological features of 60 invasive breast tumors imprinted for FISH analysis.
(XLS)

Table S2 Supporting Information Table S1: FISH signal patterns observed using the 8p21- and 8q22-specific BAC probes RP11-177H13 (labelled red, R) and RP11-10G10 (labelled green, G), respectively,to assess copy number status in 60 breast tumour touch preparations. The number of nuclei scored with the various signal patterns is indicated. Cases with a total cell count <50 are highlighted in black.
(XLS)

Table S3 Clinical follow-up data for 63 breast cancer patients.
(XLS)
