# Supplementary material for: Dual-Color Fluorescence In Situ Hybridization Reveals an Association of Chromosome 8q22 but Not 8p21 Imbalance with High Grade Invasive Breast Carcinoma
Source: PLoS One. 2013 Jul 25;8(7):e70790. doi: 10.1371/journal.pone.0070790 (PMC3723675; doi:10.1371/journal.pone.0070790)

**Supplementary Figure 1**

A representative examples of signal patterns observed on breast tumor touch imprint after FISH with BAC clones RP11-177H13 and RP11-10G10. A) BT7 - Nine nuclei counterstained with DAPI (blue) show different signal patterns, including one nucleus with normal signal configuration (N) with two copies of RP11-177H13 (8p21, red) and two copies of RP11-10G10 (8q22, green), four nuclei with one copy of RP11-177H13 and three copies of RP11-10G10 and four nuclei with two copies of RP11-177H13 and six copies of RP11-10G10. Signal patterns observed in BT7 are consistent with the formation of an isochromosome 8. B) BT2 – Two nuclei counterstained with DAPI show three copies of RP11-177H13 (8p21, red) and eight copies of RP11-10G10 (8q22, green). C) BT43- Two nuclei counterstained with DAPI show two copies of RP11-177H13 (8p21, red) and one copy of RP11-10G10 (8q22, green).

**A)**


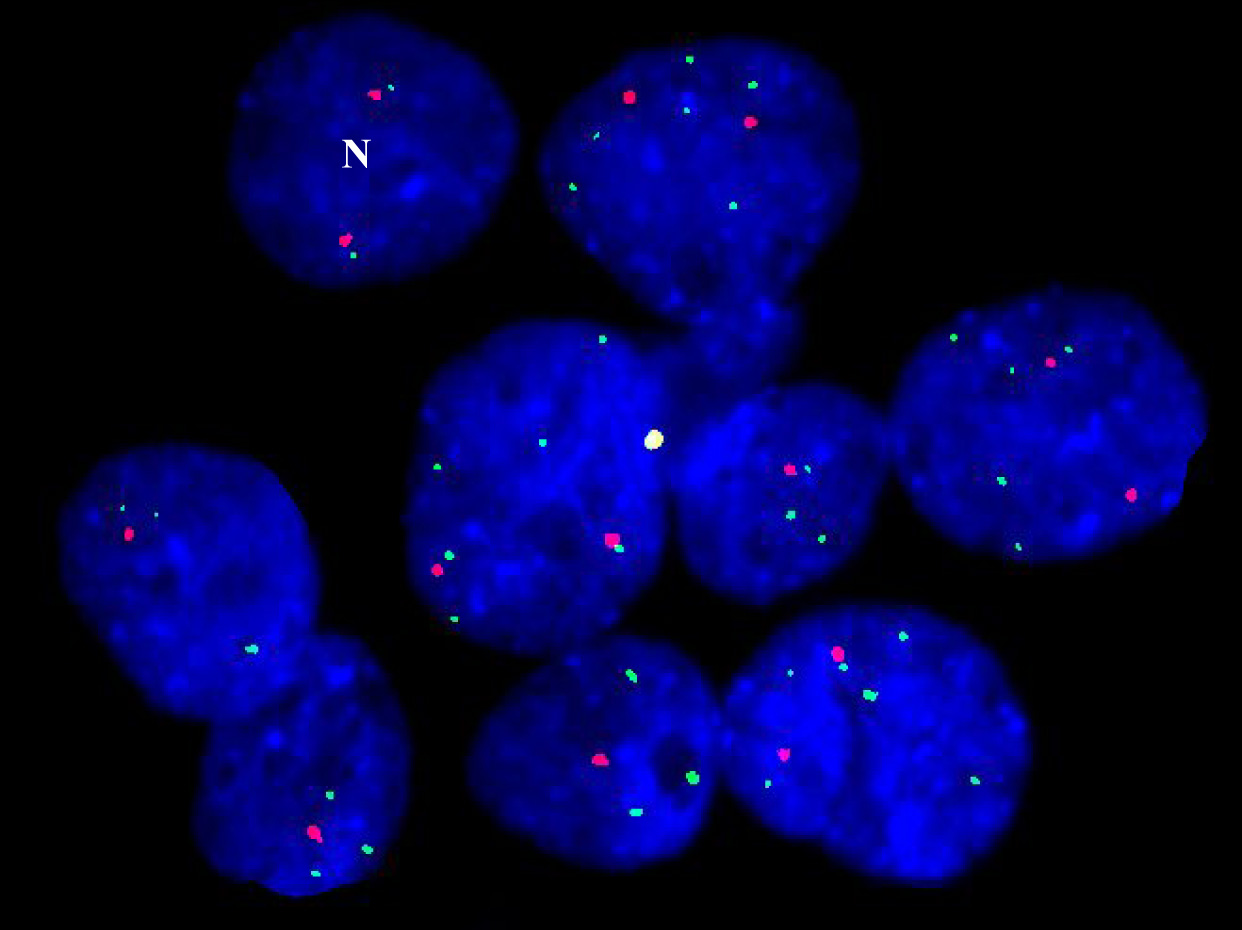


**B)**

**
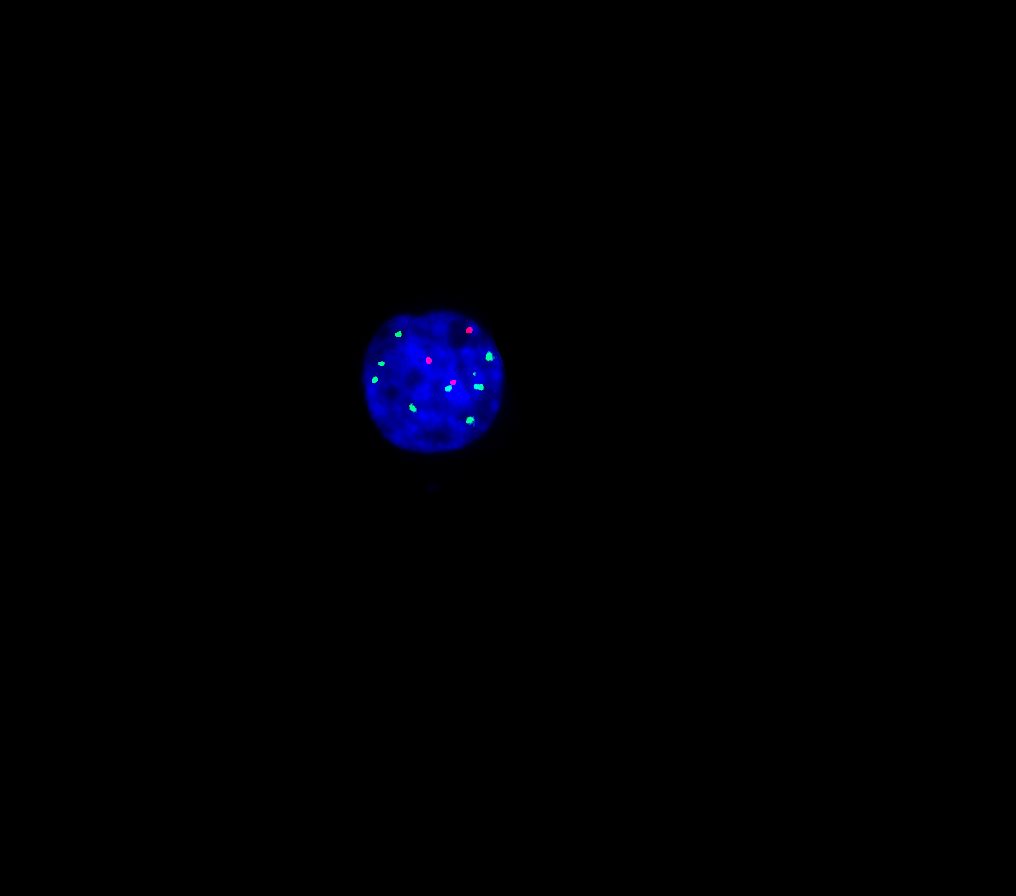

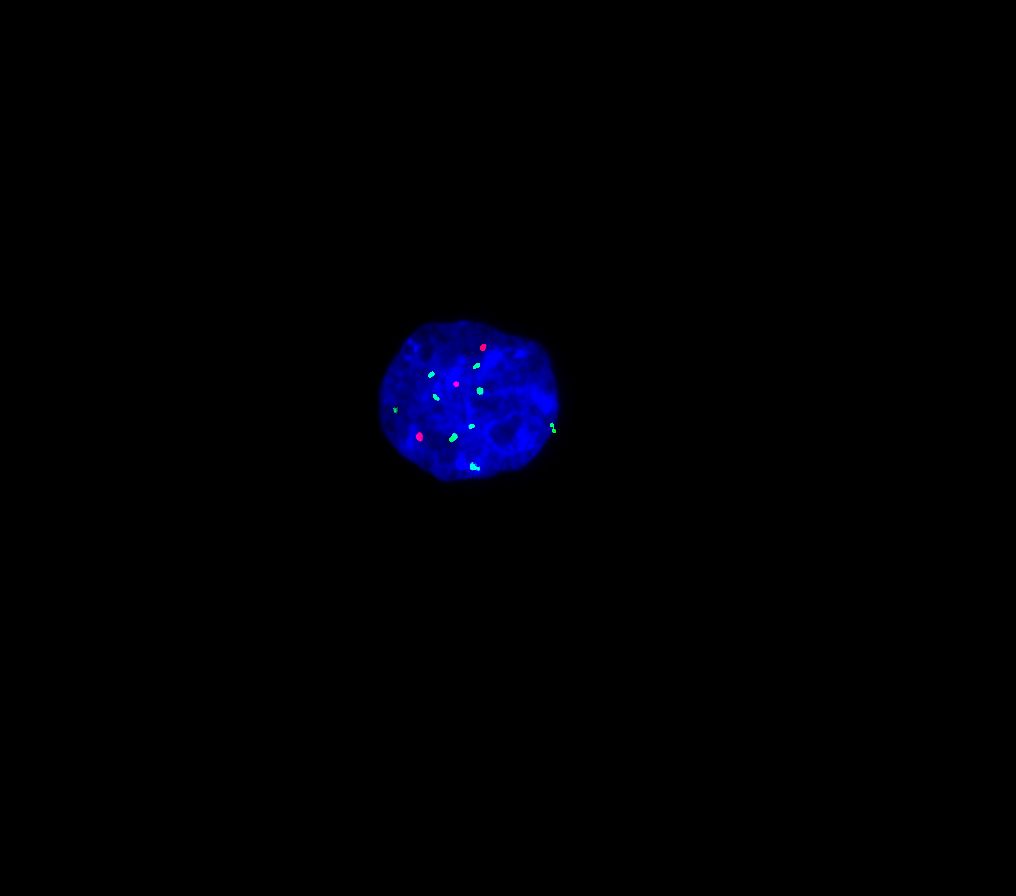
**

**C)**


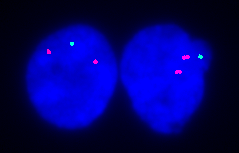

Supplement: Figure S1 — Supplementary Figure 1. (DOC) [file pone.0070790.s001.doc]
